# Report of Seven Cases of Gunshot Fracture of the Thigh, Treated Conservatively

**Published:** 1863-08

**Authors:** R. M. Lackey

**Affiliations:** Surg. 98th Ill. Vols.


REPORT OF

### SEVEN CASES OF GUNSHOT FRACTURE OF THE THIGH, TREATED CONSERVATIVELY.

By R. M. LACKEY, M. D., Surg. 98th Ill. Vols.

Conservative military surgery has had its praises and denunciations, so that if we rely upon the opinions of others as a means of forming a correct opinion ourselves, we will often be greatly puzzled to know what conclusions to arrive at in regard to the conservative treatment of certain classes of wounds. It is not my purpose at this time to advance an opinion and adduce cases in support of it; but I design merely to make a brief report of a few cases that have come under my observation with the hope that it may aid the profession in determining what *are* the advantages of conservative treatment, in the class of wounds to which the following cases belong.

Case I.—Ed. O'Neil 16th U. S. Infantry, age 40 years, has always been healthy and temperate. He was wounded at the battle of Stone River, Dec. 31, 1862. The ball entered anteriorly in the upper portion of the middle third of the left thigh, fracturing and comminuating the bone. He was removed to the field hospital near the battlefield and but little dressing used besides bandaging the limb; no erysepelas or gangrene occurred, and at this date April 20, 1863, there is firm union of the bone, and but little discharge from the wound. There is considerable angularity of the limb and two inches and a half shortening. With care he will be able to be around on crutches in about a fortnight.

Case II.—Jno. M. Whortenbury, Private 15th Reg. Vols., 18 years of age, temperate habits and tolerably good constitution.

He was wounded at Stone River, Dec. 31st, 1862, by a minnie ball which entered anteriorly in the lower third of the left thigh, producing a comminuated fracture of the bone. The treatment locally, consisted chiefly in the application of a bandage to the limb, and the constitutional treatment, in tonics, nutricious diet, &c. No erysipelas or gangrene has occurred up to this time, and there is now firm union of the bone, and if nothing unfavorable supervenes he will be up in a short time. There is shortening of two inches and a half. According to the account this boy gives, he was evidently wounded by a minnie ball at short range.

Case III.--Jas. Gilloghy, Private 21st Ill. Vols., 28 years of age of good constitution; was wounded at Stone River, Dec. 31st, 1862. The ball entered posteriorly, fracturing the thigh in the middle third. The treatment in this case was about the same as that used in the preceding ones. No erysipelas or gangrene occurred. At this date there is firm bony union and a starch bandage has been applied and the man is going around on crutches. There is but little angularity and the limb is only an inch and a half shortened. There was in this case evidently but little comminuation of the bone as there were no spiculæ came out of the wound, and the shortening is less than in the other cases. No extension was used in either of the cases.

Case IV.—Thomas Thomason, Private 15th Wis. Vols., aged 19 years; was wounded at Stone River, Tenn., Dec. 31st, 1862. The ball entered posteriorly in the middle third of the thigh, and lodged beneath the skin on the anterior portion of the thigh, a little above the knee joint. The point of fracture was in the middle third. There is still some discharge of matter from both openings, and the patient is considerably emaciated; he is in better condition now, however, than he has been at any time since he was wounded, and if he continues and improves as he has been doing, there is every reason to believe that he will recover with a tolerably useful limb. There is shortening of two inches in this case.

Case V.—Andrew Cox, Private 88th Ill. Vols., aged 32

years, was wounded at Stone River, Tenn., Dec. 31st, 1862. The ball entered anteriorly in the middle third of the thigh and emerged posteriorly two inches lower than it entered. The femur was fractured and comminuated at about the union of the middle and lower third. He was removed to Hospital No. 1 Murfreesboro, and the limb placed on a double inclined plane, where it remained until a few days ago, when a starch bandage was applied and the man is now going around on crutches. There was, I think but little splintering of the bone in this case, as there were but few spiculæ came out, and the wounds closed unusually soon. No erysipelas occurred. There is two inches shortening of the limb. This man refused a discharge from the service and insisted that he could be useful yet as an ambulance driver. Gunshot fracture of the thigh is not so fearful a wound, when it occurs in a man of the physical endurance and *pluck* of Andrew Cox.

Case VI.—John Handy, private, 101st Ohio Vols., aged 26 years, received gunshot fracture at Stone's River, Dec. 31st, 1862. The ball entered on the outer side in the lower third of the thigh, passing through and fracturing the femur. Spiculæ of bone came away with the matter discharged from the wound, for a long time. The limb was placed in straight position and but little dressing applied. There is now bony union and the wound is almost closed. This case now promises to be happy in its results.

Case VII.—Private Sanders, 36th Ills. Vols., aged 18 years, was one of the brave boys who fought so stubbornly on the "right wing" at Stone's River, and he was wounded on the memorable 31st of Dec., 1862. He says he was struck at short range and by an Enfield rifle ball, which entered anteriorly in the upper third of the left thigh. The treatment was simple: the limb placed in a straight position, without splints, and kept well cleansed. The bone is united and the boy's general condition is good; there is still some discharge from the wound, but the case bids fair to be successful. There is a shortening of about two inches and a half. A large number of spiculæ of bone have come away, showing that there was extensive comminution.

The course of procedure recommended by Guthrie in this class of wounds is seldom adopted by our Surgeons. He says (page 151, Commentaries of the Surgery of War,) "An examination by the finger in the first instance is necessary to ascertain the extent of injury to the bone, and to enable the Surgeon to remove the broken portions, as well as the ball and any extraneous substances that may be in the wound. The incisions necessarily required for this purpose in the thigh, are sometimes neglected, or the Surgeon refrains from making them from the great thickness of the muscular parts, &c." Now the incisions referred to here are, according to my observation, for some reason seldom made by our Surgeons, and I think there is no doubt but that more cases of this kind of injury could be successfully treated, if dilatation of the wound by incision was more frequently resorted to. It was once the common practice with military Surgeons to dilate, by incision, the wound made by the ball, with a view of "limiting the inflammation." John Hunter pointed out that an incision could not alter the nature of a contused wound, and only superadded another injury to the one already inflicted by the bullet, and since that time the practice has been reprobated by most Surgeons. One writer on Military Surgery, (Baudens,) calls the process "useless and barbarous," and as a means *merely* of limiting the inflammation, the operation is certainly unnecessary, but for other purposes I regard it as highly useful. In the majority of cases of this nature, death occurs from exhaustion and vitiation from long continued irritation and suppuration, the matter often burrowing among the tissues and forming cavities where it remains until it becomes exceedingly offensive and the structures around become gangrenous. I regard the operation of great importance as a remedy for those evils; the best means of lessening inflammation in a gunshot wound, and one that is in most instances successful, is by cold irrigation, water dressings, with rest, &c. The main advantages in making a free incision, enlarging either the aperture of entrance or exit, are in affording a free exit for the matter and spiculæ of bone, thus lessening the danger of exhaustion from long continued irritation, and avoiding the evil consequences attending the burrowing and retention of the discharges. These incisions should be made primarily and at whichever aperture is most dependant, provided, an incision made in the direction of the axis of the limb, at this point does not involve important structures, as blood vessels, nerves, &c. The loose spiculæ of bone, and any extraneous substances, should be removed at once and a free opening maintained to afford an easy exit for any pieces of bone or sloughs that may subsequently separate.

Patients sometimes die after the bony union has taken place and in these cases, upon examination, it will be found that the pus and blood have burrowed, for want of a free external opening, and formed cavities among the muscles, and the patient eventually dies from the extensive disease of the soft parts, after bony union is complete. When I first saw Ed. O'Niel, (Case I,) about two months and a half after he was wounded, I had but little hope of his recovery. There had been no incision made to allow the discharges to escape readily and cavities filled with pus and blood were found even among the Glutei muscles. I made a free opening and after the matter escaped injected a solution of Iodine, which was repeated for several days, and there was soon a decided improvement in the man's general condition, and the discharge became less and more healthy. Bandaging the limb is useful in preventing the infiltration of blood and pus among the tissues, but it is difficult in most cases to apply a bandage without causing the patient great suffering in moving the injured limb.

It is of great importance that the patient should have a bedstead and good mattrass to lie upon; few cases of gunshot fracture of the thigh get well in less than three months, and it requires great care to prevent the occurrence of bed-sores. The straight position I regard as the best, and but few and light dressings and splints can be applied with advantage.

I have not seen "Smith's Anterior" used, and I think there are but few cases in which it is applicable. Good diet, egg nog, whiskey toddy, wine, &c., are essential in the constitutional management.

War is not an agreeable occupation for the *many*, and particularly not for the soldier when he has the misfortune to receive a gunshot fracture of the thigh, for under the best and most careful treatment, in a majority of cases, the patient dies after days and it may be months of intense suffering.

Murfreesboro, Tenn., April, 1863.

---

[REMARKS.—The preceding cases of Surgeon Lackey are valuable, and the suggestions he makes judicious, although some of the cases may terminate fatally from presence of diseased or necrosed bone. In compound fractures of the femur from balls, unattended with division of the principal vessels, or the Sciatic nerve, I should recommend an incision to extract fragments of bone &c., which should be kept open by a tent, extension by a weight and pulley, as used by the old surgeons, and revived by Dr. Buck of the N. Y. Hospital. I used this treatment in the case of Capt. Shipman, 1st Wis. Cavalry, wounded at Cape Girardeau, by a pistol ball on the outside of the femur below the trochanter minor, and removed 17 fragments, some of them more than three inches long. Four weeks after the operation he was doing well.

The injection of a weak solution of Iodine as an antidote to the effect of absorption is important. D. B.]

